# Associations between sleep problems and emotional/behavioural difficulties in healthy children and adolescents

**DOI:** 10.1186/s12887-023-04487-z

**Published:** 2024-01-05

**Authors:** Theresa Fulfs, Tanja Poulain, Mandy Vogel, Kolja Nenoff, Wieland Kiess

**Affiliations:** 1 LIFE Leipzig Research Center for Civilization Diseases, Leipzig University, Philipp-Rosenthal-Strasse 27, 04103 Leipzig, Germany; 2 Department of Women and Child Health, Hospital for Children and Adolescents and Center for Pediatric Research (CPL), Leipzig University, Liebigstrasse 20a, 04103 Leipzig, Germany; 3 Medical Department for Hematology, Cell Therapy and Hemostaseology, Leipzig University, Liebigstrasse. 20/22, 04103 Leipzig, Germany

**Keywords:** Children, Adolescents, Sleep problems, SDQ, CSHQ

## Abstract

**Objective:**

This study aimed to (1) characterise sleep disturbances and emotional/behavioural difficulties among healthy German children and adolescents aged 3 to 13 years, (2) examine the association between parent-reported sleep problems and emotional/behavioural difficulties, (3) point out possible relations between specific kinds of sleep disturbances and different behavioural difficulties.

**Methods:**

Data were collected between 2011 and 2015 within the LIFE Child study in Germany. The sample included 1101 3- to 13-year-old children and adolescents. Information on sleep disturbances—assessed via the Children’s Sleep Habits Questionnaire (CSHQ), emotional/behavioural difficulties—assessed via the Strengths and Difficulties Questionnaire (SDQ), and socioeconomic status was provided by participants’ parents. Multiple regressions were applied to analyse the associations between general and specific sleep disturbances (independent variables) and emotional/behavioural difficulties (dependent variables).

**Results:**

The total CSHQ score was positively associated with the total SDQ score and all SDQ subscales (emotional problems, conduct problems, hyperactivity/inattention, peer relationship problems). Most of the CSHQ subscales were related to SDQ subscale scores, except for a few non-significant relations with hyperactivity/inattention and conduct problems. The CSHQ total score, daytime sleepiness, sleep duration and parasomnias showed the strongest associations with the SDQ total score.

**Conclusion:**

This study confirms an association between children’s and adolescents’ sleep habits and psychological health. We were able to demonstrate the association between sleep problems and emotional/behavioural difficulties in a large sample of healthy participants. In particular, we observed a significant relation between parasomnias and hyperactive/inattentive behaviour as well as a significant association between emotional problems and sleep problems, especially daytime sleepiness, sleep anxiety and parasomnias.

## Introduction

Healthy sleep habits are an important aspect of healthy physical and mental development for children and adolescents [1–4]. In a large German sample, 19.5% of participating children reported general sleep problems [5]. This is comparable with another German study, in which 22.6% of children and 20.0% of adolescents reported problematic amounts of sleep-related difficulties [6]. The aim of this study was thus to identify possible associations between such sleeping difficulties and emotional/behavioural difficulties among healthy German children and adolescents.

Almost 20 years ago, comparable studies were able to show a connection between sleep problems and externalising symptoms as well as psychosocial and behavioural problems in children and adolescents [7, 8]. However, given the changes in socio-cultural conditions since then, it is worthwhile to replicate these observations in the present day. In particular, children’s and adolescents’ increasing media consumption in the last decades, especially the expanding use of mobile devices, should be mentioned in this context [9, 10]. According to previous studies, mutual dependencies between electronic media use and psychological health can be assumed [11, 12]. In addition, a connection between increasing media consumption and problematic sleeping behaviour has been described [13–15]. Further changes in the last decades, e.g., related to parenting [16–18], mental health in general [19–21], or academic pressure [22, 23], might also have changed the relationship between sleep and behavioural difficulties.

Prior investigations showed that sleep problems in children and adolescents affect their daily functioning and are associated with the development of various emotional and behavioural difficulties, such as anxiety, depression, inattention, decreased concentration as well as psychosocial problems [2, 24–29]. There is evidence for a strong mutual association between the development of sleep problems and difficulties in regulating emotions and aggression. Sleep problems and these regulatory difficulties are strongly related in their development over time, with the persistence of one problem greatly increasing the risk of the other [24]. One can assume a bi-directional relationship between sleep deprivation and deficiencies in executive functioning, regulation of impulsivity and emotional control among children [1, 24, 30]. Further results of a longitudinal study confirmed that sleep problems have a persistent negative effect on children’s emotional regulation, which may lead to ongoing sleep problems and poorer attentional regulation over time [31]. Additionally, adolescents show higher prevalence of mental health issues related to emerging sleep problems such as short sleep duration, later bedtime and poor sleep hygiene [4]. Similar results were also obtained in a German-language study, in which sleep disorders significantly increased the risk of psychological problems, including both hyperactivity and excessive emotional distress [32].

Studies examining the association between sleep problems and behaviour have typically focused on parasomnias such as nightmares or sleepwalking. Parasomnias are thought to reflect underlying stress or anxiety in children, but a limitation of many studies has been the failure to control for confounds also known to be associated with stress and anxiety, such as female gender in post-pubertal children [33–36] and low SES in general [37–39]. Therefore, this study will investigate the possible association between parasomnias and emotional/behavioural difficulties, controlling for the variables sex and SES.

The co-occurrence of parasomnias (e.g., nightmares) and emotional/behavioural problems has been the subject of numerous studies. In this regard, Li et al. observed that frequent nightmares in children are associated with emotional and behavioural problems (such as hyperactivity, frequent temper outbursts/mood disturbance) and that affected children more often experienced comorbid insomnia [40]. The results of a different long-term study confirm these findings by revealing a high incidence of psychopathological symptoms in children with chronic nightmares [41]. Accordingly, for this study, special attention was paid to the possible link between parasomnias and emotional/behavioural difficulties among children and adolescents.

Given the assumed stable nature of sleep problems, children affected with general sleep problems at a young age have a higher risk of aggravated symptoms throughout childhood and adolescence [42]. Furthermore, there is evidence that especially recurrent nightmare experience during childhood may lead to an increased vulnerability to psychological problems in adulthood [43].

Based on the relationships described above and the corresponding importance of healthy sleep, especially for the psychosocial development of children and adolescents, the current study’s aim was to investigate possible associations between sleep disturbances and behavioural difficulties among healthy German children and adolescents aged 3 to 13. We hypothesised that children with sleep difficulties would also show more emotional/behavioural problems. Another aim was to investigate whether existing sleep problems in a specific area (e.g., insomnia, parasomnia) show associations with emotional/behavioural problems. Therefore, several different sleep-related difficulties were assessed and possible associations between specific emotional and behavioural difficulties were analysed.

In contrast to other studies, which mostly considered smaller and disease-related sample, the present study is based on a large cohort of children without related clinical diagnoses. We assume that the association between sleep problems and emotional/behavioural difficulties, previously described in clinical cohorts, can also be demonstrated in a large cohort of healthy children and adolescents.

## Methods

### Participants

All data were collected between 2011 and 2015 within the LIFE Child study in Leipzig, Germany [44]. The LIFE Child study is a longitudinal child cohort study which aims to monitor healthy child development as well as the development of lifestyle diseases. The study was designed in accordance with the Declaration of Helsinki and approved by the ethics committee of the University of Leipzig (Reg. No. 264–10-19,042,010). Study participants were recruited via advertisements at different institutions (hospitals, clinics, public health centres, schools), through media (internet, radio, television) or by word of mouth. Criteria for inclusion in the present study were complete information on sleep disturbances (CSHQ) and behavioural difficulties (SDQ), both reported by parents, as well as information on socioeconomic status. Children treated with psychotropic medication were excluded from the analysis (*n* = 44). The final sample comprised 1101 children and adolescents (557 boys, 544 girls, mean age = 7.44). Informed written consent was obtained from all parents.

### Measures

#### Psychological health

Children’s behavioural difficulties were assessed with the Strengths and Difficulties Questionnaire (SDQ), which is a standard instrument measuring behavioural difficulties and strengths of children and adolescents. It consists of 5 different scales, namely emotional problems, conduct problems, hyperactivity/inattention, peer relationship problems and prosocial behaviour. Each SDQ subscale comprises five items. Sum scores on each scale range from 0 to 10 points, with higher scores indicating more behavioural difficulties or strengths. For the following analysis, only the problem scales of the SDQ (all scales except prosocial behaviour) were considered. These four subscales are added together to obtain a total difficulties score ranging from 0 to 40. Cronbach’s alpha for the individual scales were 0.91 (hyperactivity/inattention), 0.71 (emotional problems), 0.60 (conduct problems), and 0.42 (peer relationship problems). We used the validated German parent version of the SDQ (SDQ-DE) [45].

#### Sleep

To evaluate children’s sleep behaviour, parents completed the Children’s Sleep Habits Questionnaire (CSHQ), which was developed as a screening tool for sleep disturbances in children aged 4 to 10 years. Previous studies have extended the original age range to over 10 [46–49] and under 4 years [50]. The CSHQ is a retrospective, 45-item questionnaire in which parents are asked to recall sleep behaviours occurring over a “typical” recent week. 33 different items are conceptually grouped into 8 subscales (bedtime resistance, sleep onset delay, sleep duration, sleep anxiety, night wakings, parasomnias, sleep disordered breathing, daytime sleepiness). A higher score indicates more sleep difficulties. The total sleep disturbance score is calculated as the sum of all items [51]. In this study, we used the German version of the Children’s Sleep Habits Questionnaire (CSHQ-DE), which showed an internal consistency of α = 0.68 and a retest–reliability of *r* = 0.76 in previous investigations [52]. In this study, Cronbach’s alpha for the individual scales were 0.75 (sleep duration), 0.68 (bedtime resistance), 0.62 (daytime sleepiness), 0.59 (night wakings), 0.55 (sleep anxiety), 0.46 (sleep disordered breathing), and 0.45 (parasomnias). The sleep onset delay scale consists of only one item.

### Covariates

In this study, we considered age, sex, and socioeconomic status (SES) as covariates. SES was measured with an index based on the educational level (general education and vocational education) and occupational status of the mother and father, as well as monthly household income after taxes. This information was provided by the parents of the participating children. The SES index varies between 3 and 21, with higher scores indicating a higher SES. Children were assigned to three SES groups: *lower social class* (index between 3 and 8.4), *intermediate social class* (index between 8.5 and 15.4), or *higher social class* (index between 15.5 and 21) [53].

### Statistical analyses

Statistical analyses were performed using the Statistical Package for the Social Sciences, version 24.0 (SPSS 24.0, SPSS Inc., Chicago, IL, USA). We used multiple linear regression models to examine the associations between sleep problems and behavioural difficulties. The independent variables were CSHQ scores, while SDQ scores were included as dependent variables. We adjusted the model for sex, age and SES. In the case of a significant association in the broader scales (total SDQ score and total CSHQ score), more detailed analyses based on the subscales of the CSHQ (sleep anxiety, night waking, daytime sleepiness, sleep duration, sleep disordered breathing, parasomnias, bedtime resistance, sleep onset delay) and SDQ (hyperactivity/inattention, emotional problems, conduct problems, peer relationship problems) were conducted. These detailed analyses investigated how specific types of sleep disturbances related to behavioural difficulties in specific areas. The multiple regression models included scores on the individual subscales of the CSHQ as independent variables and the subscales of the SDQ as dependent variables. To counteract problems of multiple significance testing, we used the Benjamini–Hochberg procedure to control for the false discovery rate (FDR) in independent tests [54]. A *p*-value of less than 0.05 after this adjustment was considered statistically significant. Strengths of associations were described by unstandardised regression coefficients β.

We tested each and every parameter for violations of the normality assumption using Shapiro–Wilk test. Furthermore, we looked for influential outliers using a qqplot. We did not find any statistically relevant outliers.

## Results

### Descriptives

Table 1 summarises information on behavioural difficulties, sleep problems and sociodemographic characteristics among the study participants. The analysed cohort consisted of 1101 children and adolescents, including 544 females (49.4%) and 557 males (50.6%). The mean age of participants was 7.44 years. SES was rather high, with 31% of participants assigned to the *higher social class*, 60.3% assigned to the *intermediate social class * and 8.7% assigned to the *lower social class*. CSHQ/SDQ total and subscores for all children are summarised in Table 1. The mean CSHQ total score in the sample was 42.55 (SD = 5.77 [CI 95% 42.21 – 42.89]). The mean SDQ difficulties score was 9.28 (SD = 5.57 [CI 95% 8.95 – 9.61]).

**Table 1:** Characteristics of the study sample (aged 3 to 13 years), including sociodemographic characteristics, sleep problems (CSHQ), and behavioural difficulties (SDQ); (*n* = 1101)

	Mean (SD) | n (%)
Sex	
Male	557 (50.6)
Female	544 (49.4)
Age (yrs.)	7.44 (3.13)
3 – 4	269 (24.4)
5 – 7	283 (25.7)
8 – 10	309 (28.1)
11 – 13	240 (21.8)
SES	13.42 (3.22)
Lower	96 (8.7)
Intermediate	664 (60.3)
Higher	341 (31.0)
SDQ scales	
Total difficulties score	9.28 (5.57)
Hyperactivity/inattention	3.66 (2.43)
Emotional problems	2.14 (2.06)
Conduct problems	2.07 (1.64)
Peer relationship problems	1.41 (1.71)
CSHQ scales	
Total sleep disturbance score	42.55 (5.77)
Sleep anxiety	4.86 (1.42)
Night wakings	3.71 (1.17)
Daytime sleepiness	12.33 (2.79)
Sleep duration	3.88 (1.34)
Sleep disordered breathing	3.30 (0.73)
Parasomnias	8.24 (1.45)
Bedtime resistance	7.03 (1.79)
Sleep onset delay	1.57 (0.77)

*SD* Standard deviation, *SES* Socioeconomic status, *CSHQ* Children’s Sleep Habits Questionnaire, *SDQ* Strength and Difficulties Questionnaire

### Associations between parent-reported sleep disturbances and emotional/ behavioural difficulties

**Fig. 1:**
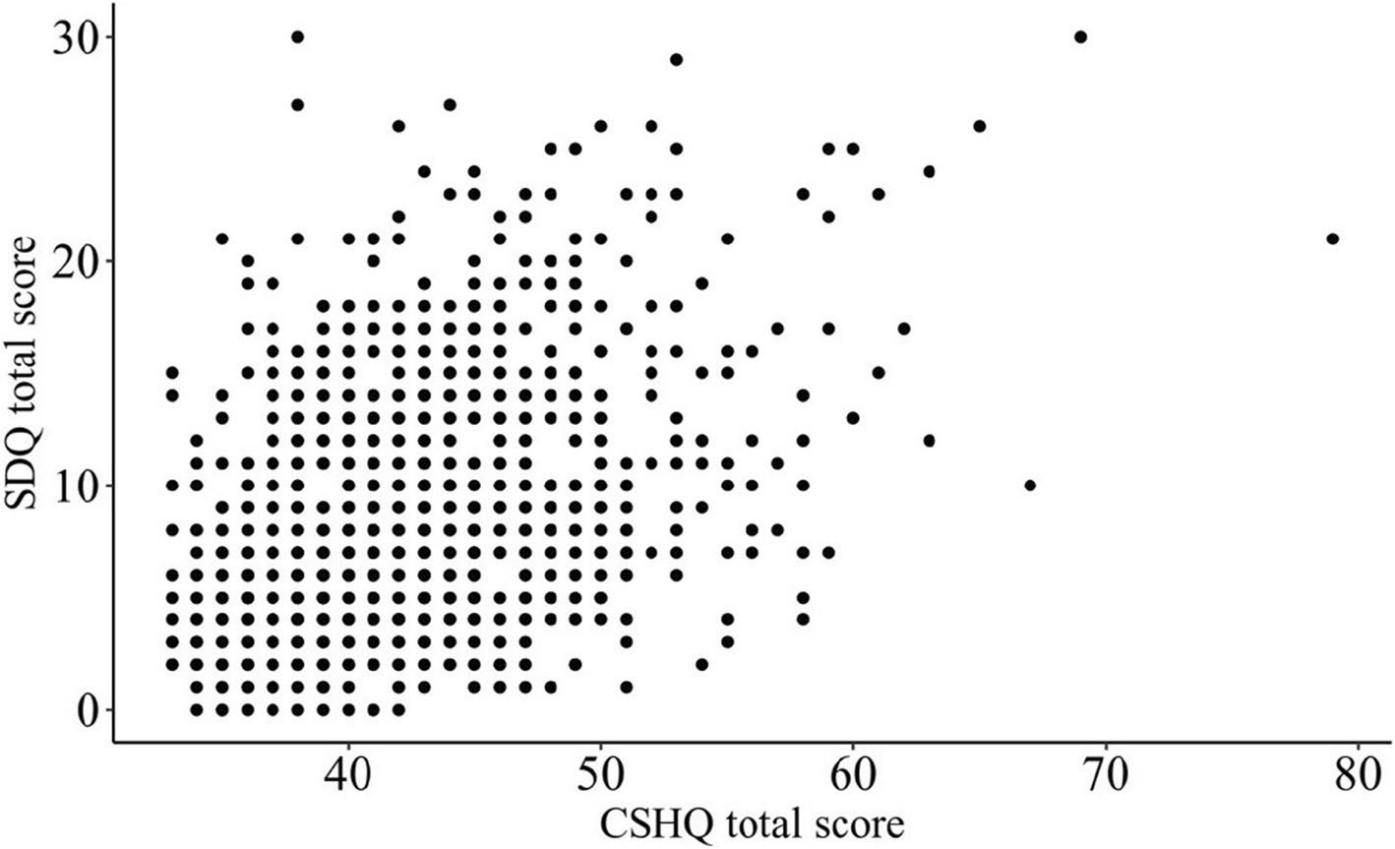
Significant association (± 95% CI) between CSHQ total score and SDQ total difficulties score (adjusted for child age, sex, SES)

We initially tested whether sleep disturbances are related to increased behavioural difficulties. Table 2 displays associations between each of the CSHQ domains and total behavioural difficulties score. According to this basic analysis, a higher total sleep disturbance score on the CSHQ was significantly associated with a higher total difficulties score on the SDQ (β = 0.363, *p* =  < 0.001) (see Fig. 1). This relation holds for the specific types of sleep disturbances, with sleep anxiety (β = 0.710, *p* =  < 0.001), night wakings (β = 0.674, *p* =  < 0.001), daytime sleepiness (β = 0.512, *p* =  < 0.001), sleep disordered breathing (β = 0.996, *p* =  < 0.001), parasomnias (β = 0.970, *p* =  < 0.001), bedtime resistance (β = 0.355, *p* =  < 0.001), sleep onset delays (β = 1.393, *p* =  < 0.001) and a reduced sleep duration (β = 1.037, *p* =  < 0.001) all significantly associated with increased emotional/ behavioural difficulties.

**Table 2:** Associations between sleep disturbances and emotional/behavioural difficulties

	Dependent variable														
	**SDQ total difficulties score**			**Hyperactivity/inattention**			**Emotional problems**			**Conduct problems**			**Peer relationship problems**		
Independent variable	*β *(95 % CI)	*P*	*Partial R*^*2*^	*β *(95 % CI)	*P*	*Partial R*^*2*^	*β *(95 % CI)	*P*	*Partial R*^*2*^	*β *(95 % CI)	*P*	*Partial R*^*2*^	*β *(95 % CI)	*P*	*Partial R*^*2*^
**CSHQ total score**	.363 (.277-.449)	<.001	.138	.100 (.061-.140)	<.001	.056	.126 (.094-.159)	<.001	.123	.071 (.044-.097)	<.001	.060	.065 (.037-.094)	<.001	.048
Age	.075 (-.083-233)	.118	.002	-.099 (-.172- -.027)	<.001	.016	.135 (.075-.195)	<.001	.041	-.041 (-.695- -.082)	.007	.006	.080 (.028-.132)	<.001	.021
Sex	-1.168 (-2.148- -.188)	<.001	.011	-.702 (-1.151- -.254)	<.001	.021	.177 (-.195-.548)	.117	.002	-.389 (-.695- -.082)	<.001	.014	-.253 (-.576-.070)	.010	.005
SES	-.398 (-.551- -.245)	<.001	.052	-.141 (-.211- -.071)	<.001	.034	-.087 (-.145- -.029)	<.001	.018	-.085 (-.133 --.037)	<.001	.027	-.085 (-.136- -.035)	<.001	.025
**Sleep anxiety**	.710 (.329-1.091)	<.001	.031	.082 (-.087-.250)	.111	.002	.403 (.264-.543)	<.001	.072	.066 (-.049-.181)	.059	.003	.159 (.039-.279)	<.001	.016
Age	.062 (-.110-.234)	.232	.001	-.115 (-.191- -.038)	<.001	.021	.146 (.083-.209)	<.001	.047	-.051 (-.103-.001)	.001	.009	.081 (.027-.135)	<.001	.021
Sex	-1.218 (-2.265- -.172)	<.001	.012	-.736 (-1.199- -.273)	<.001	.023	.186 (-.197-.569)	.109	.002	-.411 (-.728- -.095)	<.001	.016	-.257 (-.586-.072)	.010	.006
SES	-.414 (-.578- -.250)	<.001	.056	-.151 (-.224- -.079)	<.001	.039	-.084 (-.144- -.024)	<.001	.017	-.092 (-.141- -.042)	<.001	.032	-.087 (-.138- -.035)	<.001	.026
**Night wakings**	.674 (.207-1.141)	<.001	.019	.204 (.000-.409)	.001	.009	.203 (.027-.379)	<.001	.012	.098 (-.042-.238)	.021	.005	.169 (.023-.315)	<.001	.012
Age	.058 (-.117-.233)	.277	.001	-.102 (-.179- -.026)	<.001	.016	.126 (.060-.191)	<.001	.034	-.047 (-.100-.005)	.003	.008	.082 (.027-.137)	<.001	.021
Sex	-1.264 (-2.317- -.211)	<.001	.013	-.727 (-1.188- -.266)	<.001	.022	.139 (-.257-.535)	.246	.001	-.412 (-.728- -.095)	<.001	.016	-.265 (-.595-.064)	.008	.006
SES	-.457 (-.621- -.293)	<.001	.069	-.158 (-.229- -.086)	<.001	.043	-.107 (-.169- -.046)	<.001	.028	-.096 (-.145- -.047)	<.001	.035	-.096 (-.148- -.045)	<.001	.033
**Daytime sleepiness**	.512 (.327-.696)	<.001	.065	.127 (.045-.210)	<.001	.021	.193 (.124-.262)	<.001	.068	.109 (.053-.165)	<.001	.034	.082 (.023-.141)	<.001	.018
Age	-.067 (-.233-.098)	.179	.001	-.137 (-.211- -.063)	<.001	.031	.084 (.022-.145)	<.001	.016	-.070 (-.120- -.019)	<.001	.017	.056 (.003-.109)	.001	.010
Sex	-1.345 (-2.369- -.321)	<.001	.015	-.751 (-1.208- -.294)	<.001	.024	.115 (-.269-.498)	.323	.001	-.423 (-.734- -.113)	<.001	.017	-.285 (-.613-.043)	.004	.007
SES	-.458 (-.618- -.299)	<.001	.070	-.158 (-.229- -.086)	<.001	.043	-.108 (-.168- -.048)	<.001	.029	-.097 (-.145- -.048)	<.001	.036	-.096 (-.147- -.045)	<.001	.033
**Sleep duration**	1.037 (.651-1.424)	<.001	.061	.309 (.137-.481)	<.001	.029	.269 (.122-.417)	<.001	.030	.269 (.153-.386)	<.001	.048	.190 (.066-.313)	<.001	.022
Age	-.070 (-.236-.096)	.165	.002	-.141 (-.214- -.067)	<.001	.032	.090 (.026-.153)	<.001	.018	-.073 (-.123- -.023)	<.001	.019	.054 (.001-.107)	.001	.010
Sex	-1.390 (-2.416- -.363)	<.001	.016	-.765 (-1.220- -.309)	<.001	.025	.103 (-.288-.495)	.383	.001	-.435 (-.744- -.127)	<.001	.018	-.293 (-.621-.034)	.003	.007
SES	-.446 (-.606- -.286)	<.001	.066	-.154 (-.225- -.083)	<.001	.042	-.104 (-.165- -.043)	<.001	.026	-.094 (-.142- -.046)	<.001	.034	-.094 (-.145- -.043)	<.001	.031
**Sleep disordered breathing**	.996 (.271-1.722)	<.001	.017	.380 (.063-.697)	<.001	.013	.273 (.001-.546)	.001	.009	.114 (-.103-.332)	.083	.003	.229 (.001-.456)	.001	.009
Age	.007 (-.162-.177)	.885	.000	-.116 (-.190- -.042)	<.001	.022	.110 (.046-.174)	<.001	.028	-.055 (-.106- -.004)	<.001	.011	.069 (.016-.122)	<.001	.016
Sex	-1.296 (-2.349- -.243)	<.001	.014	-.733 (-1.193- -.273)	<.001	.023	.129 (-.268-.525)	.285	.001	-.418 (-.734- -.101)	<.001	.016	-.274 (-.604-.055)	.006	.006
SES	-.427 (-.592- -.262)	<.001	.060	-.146 (-.218- -.074)	<.001	.037	-.099 (-.161- -.037)	<.001	.024	-.092 (-.142- -.043)	<.001	.032	-.089 (-.141- -.038)	<.001	.028
**Parasomnias**	.970 (.608-1.332)	<.001	.061	.362 (.203-.521)	<.001	.045	.311 (.174-.447)	<.001	.046	.152 (.042-.263)	<.001	.017	.145 (.029-.261)	<.001	.014
Age	.068 (-.100-.235)	.182	.001	-.094 (-.167- -.020)	<.001	.014	.130 (.067-.193)	<.001	.038	-.045 (-.096-.006)	.004	.007	.077 (.023-.261)	<.001	.019
Sex	-1.188 (-2.216- -.159)	<.001	.011	-.693 (-1.145- -.241)	<.001	.020	.165 (-.223-.554)	.161	.002	-.399 (-.713- -.084)	<.001	.015	-.262 (-.591-.068)	.009	.006
SES	-.406 (-.567- -.245)	<.001	.054	-.139 (-.209- -.068)	<.001	.033	-.091 (-.152- -.030)	<.001	.020	-.088 (-.137- -.039)	<.001	.029	-.088 (-.140- -.037)	<.001	.027
**Bedtime resistance**	.355 (.053-.657)	<.001	.012	.047 (-.085-.180)	.237	.001	.181 (.068-.293)	<.001	.024	.055 (-.036-.145)	.047	.003	.072 (-.022-.167)	.012	.005
Age	.028 (-.144-.201)	.587	.000	-.118 (-.193- -.042)	<.001	.022	.125 (.061-.189)	<.001	.034	-.051 (-.103-.000)	.001	.009	.073 (.019-.127)	<.001	.017
Sex	-1.284 (-2.341- -.228)	<.001	.013	-.743 (-1.206- -.280)	<.001	.023	.145 (-.248-.539)	.224	.001	-.414 (-.730- -.098)	<.001	.016	-.273 (-.604-.058)	.007	.006
SES	-.430 (-.595- -.265)	<.001	.061	-.153 (-.225- -.080)	<.001	.040	-.095 (-.156- -.033)	<.001	.022	-.092 (-.141- -.042)	<.001	.032	-.091 (-.142- -.039)	<.001	.029
**Sleep onset delay**	1.393 (.706-2.080)	<.001	.036	.524 (.223-.825)	<.001	.027	.216 (-.045-.478)	.006	.006	.390 (.185-.596)	<.001	.033	.262 (.045-.479)	<.001	.014
Age	.054 (-.116-.224)	.294	.001	-.099 (-.173- -.024)	<.001	.015	.115 (.050-.180)	<.001	.029	-.039 (-.743- -.121)	.011	.005	.077 (.045-.479)	<.001	.019
Sex	-1.375 (-2.416- -.333)	<.001	.015	-.763 (-1.219- -.307)	<.001	.025	.111 (-.286-.508)	.357	.001	-.432 (-.743- -.121)	<.001	.017	-.291 (-.620-.038)	.004	.007
SES	-.452 (-.615- -.290)	<.001	.068	-.156 (-.227- -.085)	<.001	.043	-.106 (-.168- -.044)	<.001	.027	-.095 (-.144- -.047)	<.001	.035	-.095 (-1.46- -.044)	<.001	.032

Associations are described in terms of unstandardised regression coefficients. All associations are adjusted for age, sex, and SES

### Associations between parent-reported sleep disturbances and hyperactivity/ inattention

The subsequent, more detailed analysis included the specific types of emotional/ behavioural difficulties. We first assessed the association between sleep disturbances in general and hyperactivity/inattention. Higher total sleep disturbance scores were significantly related to higher hyperactivity/inattention subscores (β = 0.100, *p* =  < 0.001). Furthermore, significant associations between night wakings (β = 0.204, *p* = 0.001), daytime sleepiness (β = 0.127, *p* =  < 0.001), sleep disordered breathing (β = 0.380, *p* =  < 0.001), parasomnias (β = 0.362, *p* =  < 0.001), sleep onset delays (β = 0.524, *p* =  < 0.001), reduced sleep duration (β = 0.309, *p* =  < 0.001) and hyperactivity/ inattention were revealed. However, as indicated by small partial *R*^2^ (0.009—0.056), these associations were rather small. There was no significant association between sleep anxiety or bedtime resistance and increased hyperactivity/inattention.

### Associations between parent-reported sleep disturbances and emotional problems

As described for the subdomain of hyperactivity/inattention, we found an association between sleep disturbances and emotional problems as well. This is exemplified by a significant association between higher total sleep disturbance scores and increased emotional problems (β = 0.126, *p* =  < 0.001). Moreover, the detailed analysis examining the specific kinds of sleep disturbances showed that higher CSHQ subscores were all significantly related to more emotional problems such as sleep anxiety (β = 0.403, *p* =  < 0.001), night wakings (β = 0.203, *p* =  < 0.001), daytime sleepiness (β = 0.193, *p* =  < 0.001), sleep disordered breathing (β = 0.273, *p* = 0.001), parasomnias (β = 0.311, *p* =  < 0.001), bedtime resistance (β = 0.181, *p* =  < 0.001), sleep onset delays (β = 0.216, *p* = 0.006) and reduced sleep duration (β = 0.269, *p* =  < 0.001). Again, the strengths of the associations were rather small (*R*^2^ ranging between 0.009 and 0.123).

### Associations between parent-reported sleep disturbances and conduct problems

In comparison to hyperactivity/inattention and emotional problems, there were fewer significant associations between conduct problems and the specific types of sleep disturbances. However, higher total sleep disturbance scores were significantly related to higher conduct problem scores (β = 0.071, *p* =  < 0.001), as were higher scores on the scales for night wakings (β = 0.098, *p* = 0.021), daytime sleepiness (β = 0.109, *p* =  < 0.001), parasomnias (β = 0.152, *p* =  < 0.001), sleep onset delays (β = 0.390, *p* =  < 0.001) and reduced sleep duration (β = 0.269, *p* =  < 0.001). Again, *R*^2^ were rather small (0.005 – 0.060). There was no significant association between sleep anxiety, sleep disordered breathing or bedtime resistance and increased conduct problems.

### Associations between parent-reported sleep disturbances and peer relationship problems

Higher total CSHQ scores (β = 0.065, *p* =  < 0.001) as well as all specific types of sleep disturbances were significantly associated with an increased likelihood of having peer relationship problems: sleep anxiety (β = 0.159, *p* =  < 0.001), night wakings (β = 0.169, *p* =  < 0.001), daytime sleepiness (β = 0.082, *p* =  < 0.001), sleep disordered breathing (β = 0.229, *p* = 0.001), parasomnias (β = 0.145, *p* =  < 0.001), increased bedtime resistance (β = 0.072, *p* = 0.012), sleep onset delays (β = 0.262, *p* =  < 0.001) and reduced sleep duration (β = 0.190, *p* =  < 0.001). *R*^2^ ranged from 0.005 to 0.048, indicating small effects.

## Discussion

The present study investigated the associations between parent-reported sleep disturbances and emotional/behavioural difficulties among a large sample of healthy German children and adolescents between 3 and 13 years of age. This study aimed to not only determine associations between sleep disturbances in general and emotional/behavioural difficulties in general, but also to assess possible associations between specific areas of sleep disturbances and emotional/behavioural difficulties, represented by subscores of the CSHQ and SDQ.

First and foremost, we want to emphasise the association observed between parent-reported sleep problems and behavioural difficulties (reflected by total CSHQ scores and total difficulties scores on the SDQ). The strongest predictors of increased emotional/behavioural problems in general were experiencing parasomnias, daytime sleepiness and reduced sleep duration. Inversely, especially emotional problems showed associations with the occurrence of sleep difficulties such as increased sleep anxiety, daytime sleepiness, and parasomnias. Similar associations were only found between parasomnias and hyperactivity/inattention as well as between reduced sleep duration and conduct problems.

The majority of our results are in line with the results of previously published, international research [55]. In a Chinese study investigating the prevalence and factors associated with sleep disturbances in school-aged children (6–14 years), emotional symptoms, conduct problems and hyperactivity were all significantly associated with registered sleep disturbances [56]. In contrast to our results, however, the authors observed no significant associations between peer relationship problems and sleep disturbances. Particularly deserving mention are the connections between emotional problems as well as hyperactivity/inattention and sleeping difficulties, which have also been highlighted in other studies [32, 57, 58].

A further aim of this study was to assess the associations between emotional/behavioural problems and specific sleep disturbances, e.g., parasomnias. In this regard, we observed the strongest association between the presence of hyperactive/inattentive behaviour and parasomnias out of all the sleep problems surveyed. In addition, we were able to identify a significant association between the occurrence of emotional problems and daytime sleepiness, sleep anxiety as well as parasomnias. Overall, these results are comparable with the findings of other studies, e.g., a Canadian study investigating the association between parasomnias like sleepwalking and sleep talking and emotional/behavioural problems in children (4–5 years). They showed that children who experienced these kinds of sleep disturbances had more anxious, depressive and oppositional problems than children without parasomnias [59]. A study conducted in Hongkong showed that frequent nightmares in children and adolescents aged 5 to 15 years are associated with emotional and behavioural problems such as hyperactivity and mood disturbances [40]. This may indicate an association between emotional/behavioural problems and specific sleep disturbances, such as parasomnias in particular, but also sleep anxiety, daytime sleepiness and a reduced sleep duration. It is important to mention that the observed associations were rather small and might not be of high clinical relevance. Given the weak internal consistency of the parasomnias scale, these findings must also be interpreted with caution.

With these observations, we undergird the general assumption that sleep deprivation, insomnia and nightmares are particularly linked to the occurrence of mental difficulties. Moreover, we were able to specify the association between emotional problems as well as hyperactivity/inattention and the occurrence of specific sleep difficulties such as daytime sleepiness, sleep anxiety and parasomnias.

Accordingly, an increased occurrence of these kinds of sleeping difficulties, even in healthy children and adolescents, may indicate the possible existence of a psychosocial/emotional stress situation, which expresses itself in the form of the mentioned sleep disorders and vice versa.

Considering the factors mentioned above, one could thus speak of a vicious cycle of mutually reinforcing sleep problems and emotional/behavioural difficulties. The termination of temporal trends in the associations between sleep disturbances and emotional/behavioural difficulties in children and adolescents is complicated by several methodological difficulties, such as the lack of comparable measures. Nonetheless, despite the socio-cultural changes mentioned earlier, the associations between sleep disturbances and emotional/behavioural problems appear to have remained stable for more than three decades [60–62].

### Strengths and limitations

Even though the CSHQ cannot provide a differentiated diagnosis of a specific sleep disorder, this questionnaire can be used as a screening instrument in everyday paediatric clinical practice to provide initial indications of sleep problems and possible accompanying psychosocial behavioural problems. Regarding the interpretation of the results, the low Cronbach’s alpha values for some of the CSHQ scales (sleep disordered breathing, parasomnias) and one of the SDQ subscales (peer relationship problems) must be mentioned. The accuracy of the measurements of these constructs may be limited. Corresponding associations should therefore be assessed with caution.

Because of the study’s cross-sectional design, we cannot draw long-term conclusions based on the presented observations and co-occurrence of symptoms. Still, despite the lack of conclusiveness regarding causality, our results clearly suggest the benefit of clinical intervention/treatment for both isolated or simultaneously occurring sleep and/or behavioural disturbances. For children and adolescents with attention problems (e.g., at school) or emotional/psychosocial problems, it can be useful to pay attention to good sleep hygiene and to provide education and intervention regarding healthy sleep behaviour.

Since this study showed particular associations between parasomnias, daytime sleepiness, reduced sleep duration and overall behavioural/emotional difficulties, it may also make sense to pay special attention to these specific areas of sleep problems. In further studies in this field, it could therefore be useful to look specifically at the connection between parasomnias and possibly simultaneously occurring sleep disorders and associated emotional/behavioural problems in children and adolescents.

It should be mentioned that due to the high socioeconomic status of the participants, the available data are not representative of the general population. Furthermore, the present study is limited by its reliance on subjective/parent-reported measures. Parents’ assessments of children’s sleep habits show acceptable results when it comes to the occurrence of symptoms but are less reliable than actigraphic assessments in correctly estimating sleep onset or duration [63]. Therefore, there is a potential rater bias. Although this study controls for numerous confounding factors that might affect children’s sleep and mental health status, residual confounding is possible.

## Conclusion

This study confirms an association between children’s and adolescents’ sleep habits and their psychological health. We were able to demonstrate the co-occurrence of sleep problems and emotional/behavioural difficulties in a large sample of healthy participants. Particular attention should be paid to the relationship between parasomnias and emotional/behavioural difficulties, as we observed a significant relation between the occurrence of parasomnias and hyperactive/inattentive behaviour as well as a significant association between emotional problems and sleep problems, especially daytime sleepiness, sleep anxiety and parasomnias.

## Data Availability

The data sets on which the present study is based are not publicly accessible, since the publication of data is not covered by the informed consent given by study participants. Potentially sensitive information is collected in the context of the LIFE Child study; therefore, the data protection concept requires that all researchers interested in accessing the data sign a project agreement. Researchers interested in gaining access to the data collected in the LIFE Child study may contact the committee on data use and access (dm@life.uni-leipzig.de).
